# PET performance evaluation of a preclinical digital PET/MRI insert

**DOI:** 10.1186/2197-7364-1-S1-A3

**Published:** 2014-07-29

**Authors:** David Schug, Christoph Lerche, Peter Dueppenbecker, Pierre Gebhardt, Benjamin Goldschmidt, Andre Salomon, Jakob Wehner, Bjoern Weissler, Fabian Kiessling, Volkmar Schulz

**Affiliations:** Department of Physics of Molecular Imaging Systems, Institute for Experimental Molecular Imaging, RWTH Aachen University, Aachen, Germany; Philips Research, Eindhoven, X-Ray Imaging Systems, Kragujevac, The Netherlands; Division of Imaging Sciences and Biomedical Engineering, King’s College London, London, UK; Philips Research Laboratories, Aachen, Molecular Imaging Systems, Kragujevac, Germany; Institute for Experimental Molecular Imaging, RWTH Aachen University Hospital, Aachen, Germany

In recent years, several groups have presented combined PET/MRI devices for clinical and preclinical research. Using new technologies like SiPMs, the performance of the systems improved over the years allowing a detailed exploration of the relatively new field of hybrid imaging modality. First presented at the NSS/MIC 2012, our group built a PET/MRI insert based on PDPC’s digital SiPMs (DPC). It is to date the only combined PET/MRI system using this technology which allows the performance evaluation of DPCs on system level. The Hyperion-II^D^ PET/MR insert consists of 10 PET modules. Each PET module comprises 6 detector stacks whereas one of them consists of an interface board with local FPGA and a sensor tile. The latter employs a 4x4 array of sensor dies, each consisting of 2x2 DPCs. For photon detection, a 30x30 LYSO crystal array (1mm pitch, 12mm length) is glued using an intermediate 2mm light guide on top of each sensor tile. Measurements were performed using ^22^Na point sources (point like, activity = 1.5MBq) for different scenarios: the position dependence of the mentioned parameter was tested by moving a point source along the z axis. Different sensor configurations (trigger settings, validation thresholds, etc.) as well as temperature conditions were applied to characterize the influence of these parameters on the performance. We use an Anger algorithm and a Maximum Likelihood algorithm for crystal identification.

We measure 12.3% (FWHM) energy resolution and a CRT of about 470ps and 520ps (FWHM) for trigger schemes 2 and 3. No dependence on the axial position is observed and no difference between the measurement inside and outside the magnetic field occurs. We measure for both B_0_ field scenarios an effective sensitivity of about 3.1%.

Only minor effects due to the operation inside the B_0_ field can be observed. More results and details about this study will be presented at the conference.

